# Exploring the impact of AI technostress on physicians’ job insecurity and performance from an empirical multi-hospital study

**DOI:** 10.1016/j.isci.2025.114394

**Published:** 2025-12-09

**Authors:** Chung-Feng Liu, Tzu-Chi Lin, Yen-Ling Ko

**Affiliations:** 1 Department of Medical Research, Chi Mei Medical Center, Tainan 710402, Taiwan; 2 Department of Nursing, Chi Mei Medical Center, Liouying, Tainan 73657, Taiwan; 3 Department of Nursing, Min-Hwei Junior College of Health Care Management, Tainan 73658, Taiwan; 4 Department of Internal Medicine, Chi Mei Medical Center, Tainan 710402, Taiwan; 5 General Education Center, Chia Nan University of Pharmacy and Science, Tainan 717301, Taiwan

**Keywords:** Health sciences, Social sciences

## Abstract

This study expands Califf’s technostress model, which explores the psychological stress caused by technology, by integrating “perceived self-esteem threat” as a key stressor driven by the growing influence of medical artificial intelligence (AI), examining its impact on physicians’ job insecurity and performance. A survey of 400 physicians from three Taiwanese hospitals (92.4% response rate) revealed the nuanced effects of AI-related technostress. Structural equation modeling (SEM), a statistical method used to test relationships between variables, showed that complexity and technology overload significantly increase job insecurity, while AI reliability, unexpectedly, also heightens it. AI self-esteem threat emerged as the most influential source of technostress. Job insecurity negatively affects job satisfaction but unexpectedly boosts job performance, suggesting a motivational response to perceived threats. The expanded model explains 44.6% of the variance in psychological reactions to AI, underscoring the critical role of self-esteem threat in shaping physicians’ well-being and performance.

## Highlights

• Incorporating self-esteem threat into technostress reveals a new physician stressor
• Multi-center survey (n = 400) links self-esteem threat with physicians’ job insecurity
• AI reliability, complexity, and overload all show significant associations with job insecurity
• Job insecurity reduces job satisfaction yet shows a positive link to self-rated performance

## Graphical abstract

**Figure:**
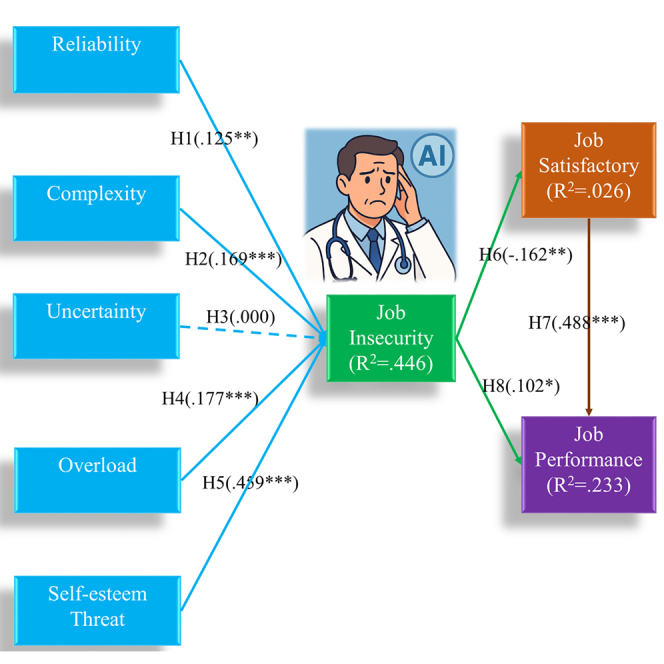

## Introduction

### Background and motivation

Artificial intelligence (AI) has become a transformative technology, significantly impacting work and lifestyle. While AI adoption has brought improvements in efficiency and decision-making, concerns about its unintended consequences persist.^1^ A review found that most AI models from 2019 to 2021 aimed to support rather than replace physicians.^2^ However, advancements in AI, including large language models (LLMs) and generative AI (GAI) in 2023, have elevated its capabilities, often matching or surpassing human performance in complex tasks, particularly in healthcare. For instance, a comprehensive review by Rajpurkar et al.^3^ highlights that in the field of medical imaging diagnostics-encompassing radiology, pathology, and ophthalmology-deep learning models can achieve diagnostic accuracy on par with, or even superior to, that of specialist physicians. These demonstrated capabilities underscore the rapid progress of AI. Besides, recent systematic evidence indicates that LLMs have been extensively applied in medicine.^4^ Susskind and Susskind^5^ argue in the future of the professions that “increasingly capable systems will transform the nature of professional work,” shifting expertise from humans to machines. These developments have raised fears of AI replacing healthcare professionals, impacting their job security and performance,^4^^,^^6^^,^^7^ particularly for physicians, whose expertise is central to their professional identity.

### Technostress as a psychological barrier in AI adoption

Despite its transformative potential, AI adoption in healthcare remains limited. The Technology Acceptance Model (TAM) suggests that perceived usefulness and ease of use influence users’ intention to adopt technology.^8^ Nevertheless, the use of information and communication technology can exert both positive and negative influences on employees’ stress and well-being.^9^ Similarly, the adoption of AI exhibits a “double-edged sword effect” on employees’ innovation behavior.^10^ This multifaceted impact is evident in the medical AI domain, where high acceptance rates among physicians do not always translate into widespread practical implementation. A case in point is a large hospital where, despite physicians’ expressed enthusiasm for an AI-based pneumonia prediction tool, actual usage fell significantly short of expectations.^11^^,^^12^ Psychological barriers, such as technostress, may hinder technology adoption across various contexts.^13^ This challenge becomes even more pronounced with “black-box” AI systems, where opacity and unpredictability may further intensify users’ stress.

Historically, the concept of technostress was introduced by Brod^13^ as a “disease of adaptation” arising from difficulties in coping with computer technologies. It was later refined by Tarafdar et al.^14^^,^^15^ in the information systems field, emphasizing how technological stressors induce psychological strain. In the healthcare context, Califf et al.^16^ extended this understanding by demonstrating that technology can have both positive and negative effects on professionals’ psychological well-being and career attitudes. With the rise of AI, technostress has been further reframed to encompass new emotional and cognitive challenges, such as self-esteem threat and anxiety over role replacement.

Technostress arises from adapting to and using technologies, often driven by system complexity, information overload, and blurred work-life boundaries. Evidence from the COVID-19 pandemic shows that new ways of working, such as remote and hybrid arrangements, have intensified technostress and reduced employees’ well-being.^17^ Physicians experience stress from electronic medical records due to system complexity and dependence on technology.^18^ Research^19^ highlights that HIT-induced stress, such as poor design and after-hours use, negatively affects physicians’ mental health despite its efficiency benefits.

In healthcare, technostress manifests as cognitive overload, dependency on complex systems, and anxiety about rapidly evolving technologies. A survey revealed that healthcare workers, while acknowledging the efficiency and accuracy of AI, reported concerns about increased workload, reduced autonomy, and shifts in job roles.^7^ These challenges foster resistance to AI adoption. The stakes are particularly high in healthcare, where technostress can compromise patient safety. Unlike other fields, healthcare technostress poses unique risks, including burnout and diminished care quality.^14^^,^^20^

Califf et al.’s technostress model^16^ provides a comprehensive framework for understanding psychological responses to technology and the career attitude. However, AI introduces unique stressors, such as perceived self-esteem threats, which occur when healthcare professionals view AI’s superior capabilities as a challenge to their expertise and professional value. These threats can undermine confidence, job satisfaction, and job security.^21^ By incorporating self-esteem threat as a new dimension, this study expands the technostress model to explore the psychological impacts of AI adoption on physicians. Understanding these barriers is essential to reducing job insecurity and promoting balanced AI integration in clinical practice.

### Research objectives and questions

Despite increasing research on technostress, empirical studies focusing on AI-specific stressors in healthcare remain limited. Prior literature has mainly examined information systems and electronic medical records, overlooking the psychological effects unique to AI, particularly the threat to professional self-esteem that arises when physicians perceive AI as challenging their expertise and value. Addressing this gap, the present study extends Califf et al.’s technostress model by incorporating self-esteem threat as a key AI-induced stressor and empirically validating its effects on physicians’ job insecurity, job satisfaction, and performance across hospitals.

The study addresses the following research questions.

1. How do different AI-induced technostressors affect physicians’ job insecurity?
2. How does AI-induced job insecurity shape physicians’ workplace experiences?

### Research insights and significance

This study aims to provide insights in three key areas.

1. Theoretical advancement: expands the Califf et al.’s technostress model by introducing perceived self-esteem threat as a key negative dimension within AI-driven work environments.
2. Policy insights: delivers actionable strategies for policymakers to mitigate the adverse impacts of AI on healthcare workers’ well-being.
3. Practical implications: provides actionable recommendations for healthcare AI stakeholders to enhance the integration of AI while minimizing job insecurity.

This research highlights AI-induced technostress, offering insights into healthcare workforce dynamics, informing policy, and guiding effective AI implementation to help professionals adapt and thrive.

### Concise hypothesis development summary

To provide a smooth transition from the theoretical background to the formal hypothesis statements presented in the subsequent “research framework and hypotheses” section, we summarize the conceptual rationale underlying the proposed relationships.

Prior literature suggests that AI reliability may inadvertently heighten physicians’ job insecurity by reducing the perceived necessity of human judgment. AI complexity increases cognitive burden and decreases confidence, thereby reinforcing insecurity. AI uncertainty, arising from the rapid evolution and unpredictability of AI systems, may create discomfort and perceived instability in the work environment. AI overload introduces additional time pressure and workflow disruptions, intensifying concerns about role stability. Moreover, when physicians perceive that AI challenges their professional expertise, self-esteem threat may arise, undermining confidence and increasing job insecurity. Job insecurity may subsequently reduce job satisfaction, while job satisfaction is generally associated with improved performance. Conversely, job insecurity may sometimes trigger compensatory behaviors aimed at protecting one’s professional role.

**Figure 1:**
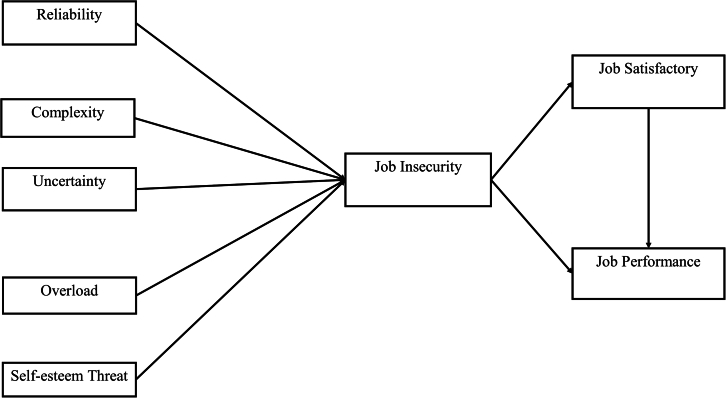
Research framework The proposed research framework illustrates the hypothesized relationships among five AI-induced technostressors—AI reliability, AI complexity, AI uncertainty, AI overload, and AI self-esteem threat—job insecurity, job satisfaction, and job performance. Arrows represent hypothesized directional paths (H1–H8).

Figure 1 illustrates the proposed research framework, while the definitions of the related variables and corresponding measurement items are detailed in Tables 1 and 2. A detailed theoretical rationale and extended hypothesis development are provided in Methods S1*: extended literature review and hypothesis development* (supplemental information), which cites references.^26^^,^^27^^,^^28^^,^^29^^,^^30^^,^^31^^,^^32^^,^^33^^,^^34^^,^^35^^,^^36^^,^^37^^,^^38^^,^^39^^,^^40^^,^^41^^,^^42^^,^^43^^,^^44^^,^^45^^,^^46^^,^^47^^,^^48^^,^^49^^,^^50^^,^^51^^,^^52^ The formal hypotheses (H1–H8) are listed in the subsequent research framework and hypotheses section.

**Table 1:** Operational definitions of constructs in the research framework

Variable	Operational Definition	Reference Source
Job Insecurity	Physicians’ subjective perception and concern about the possibility of losing their jobs in the future.	Vander Elst et al.^22^
Job Satisfaction	A positive or pleasurable emotional state resulting from physicians’ evaluation of their clinical work or experiences.	Califf et al.^16^
Job Performance	Physicians’ self-perceived overall performance in providing medical care.	Karr-Wisniewski and Lu^23^
AI Self-esteem Threat	Physicians’ feeling of their professional competence being challenged due to high dependence on AI.	Tang et al.^24^
AI Complexity	The degree to which physicians perceive the need to invest effort in understanding and learning AI usage.	Califf et al.,^16^ Ayyagari et al.^25^
AI Reliability	The degree to which physicians perceive the reliability of AI functions and performance.	Califf et al.,^16^ Ayyagari et al.^25^
AI Uncertainty	The degree to which physicians feel discomfort and uncertainty due to ongoing AI technological changes and upgrades.	Califf et al.^16^
AI Overload	The degree to which physicians feel compelled to work faster and longer due to AI integration.	Califf et al.^16^

**Table 2:** Questionnaire items for each construct

Variable	Measurement Items	Reference Source
Job Insecurity (JI)	JI1. It is possible that I will lose my job soon (or have fewer job opportunities). JI2. I am not sure if I can keep my job. JI3. I feel insecure about the future of my job.	Vander Elst et al.^22^
Job Satisfaction (JS)	JS1. I like the work I do. JS2. I feel proud of my job. JS3. I enjoy my work.	Ragu-Nathan et al.^15^
Job Performance (PR)	PR1. I think AI helps improve the quality of my work. PR2. I think AI helps improve my work efficiency. PR3. Compared to traditional methods, AI helps me accomplish more tasks. PR4. Overall, I feel I can complete my work efficiently. PR5. Overall, I feel I can complete my work with quality.	Karr-Wisniewski and Lu^23^
AI Self-Esteem Threat(SET)	SET1. I feel that the introduction of AI makes my medical skills and knowledge less significant. SET2. I feel that after using AI, my professional judgment may no longer be seen as central. SET3. In the AI environment, my clinical experience and insights may be significantly challenged.	Tang et al.^24^
AI Reliability (RE)	RE1. I believe that the AI functions in our hospital are dependable. RE2. I believe that the capabilities of our hospital’s AI are reliable. RE3. I believe that the operations (e.g., functionality, presentation) of our hospital’s AI are consistent.	Califf et al.^16^
AI Complexity (TC)	TC1. It takes me a long time to understand and use AI-assisted technologies. TC2. I do not have enough time to learn and improve my AI knowledge or skills. TC3. I find it too complicated to understand and use AI technologies.	Califf et al.^16^
AI Uncertainty (TU)	TU1. I feel that our hospital’s AI technology is always evolving (e.g., adopting new algorithms, interaction methods, etc.). TU2. I feel that the AI software in our hospital is constantly changing (e.g., adding applications in various departments, etc.). TU3. I feel that our hospital’s AI functions are frequently updated and upgraded.	Califf et al.^16^
AI Overload (TO)	TO1. I think the introduction of AI forces me to do more work. TO2. I think the introduction of AI forces me to work under very tight time constraints. TO3. I may be forced to change my work habits to adapt to AI functions.	Califf et al.^16^

## Results

A total of 433 questionnaires were distributed across three hospitals. After excluding 33 invalid responses—including those submitted by non-physicians or with missing construct items—a total of 400 valid questionnaires were obtained (92.4% response rate). Of these, 286 (71.5%) were from the medical center, 84 (21%) from the regional hospital, and 30 (7.5%) from the district hospital. Notably, many medical center physicians also support the other hospitals.

### Demographics

Table 3 shows that most respondents were male (76.5%), aged 25–35 years (39.7%), with internal medicine being the most represented specialty (28.5%). All constructs were measured using five-point Likert scales (*1 = strongly disagree to 5 = strongly agree*). To aid interpretation, mean scores were categorized as low (<3.0), moderate (3.0 ≤ mean <3.5), and high (≥3.5).

**Table 3:** Demographic characteristics of respondents (*n* = 400)

Description	Respondents (*n* = 400)
Gender, n (%)	
Female	93 (23.3%)
Male	306 (76.5%)
N/A	1 (0.2%)
Age, n (%)	
<25	2 (0.5%)
25–35	159 (39.7%)
35–45	115 (28.7%)
45–55	81 (20.3%)
55–65	41 (10.3%)
>65	2 (0.5%)
Position, n (%)	
Resident physician	132 (33.0%)
Attending physician	268 (67.0%)
Department, n (%)	
Internal medicine	114 (28.5%)
Surgery medicine	101 (25.3%)
Gynecology and pediatrics	70 (17.5%)
Emergency and critical care	22 (5.5%)
Medical imaging	35 (8.7%)
Others	58 (14.5%)
Education level, n (%)	
Bachelor’s degree	335 (83.8%)
Master’s degree	43 (10.7%)
Ph.D. degree	22 (5.5%)
Variables, mean (SD)	
Job insecurity	2.7 (0.9)
Job satisfaction	4.0 (0.7)
Job performance	3.9 (0.6)
Self-esteem threat	2.8 (0.8)
Complexity	3.3 (0.8)
Reliability	3.5 (0.8)
Uncertainty	3.3 (0.7)
Overload	3.2 (0.7)

All constructs measured on five-point Likert scales: mean <3.0 = low; 3.0 ≤ mean <3.5 = moderate; mean ≥3.5 = high.

Accordingly, survey results indicated low AI-related self-esteem threat and job insecurity (mean <3.0), moderate technostress dimensions including complexity, uncertainty, and overload (mean <3.5), and high AI reliability, job satisfaction, and performance (mean ≥3.5).

### Measurement model assessment

All constructs demonstrated satisfactory reliability and validity. Cronbach’s alpha values exceeded 0.7, and composite reliability (CR) values were above 0.7, indicating good internal consistency. Convergent validity was confirmed as all standardized factor loadings ranged from 0.676 to 0.937, surpassing the 0.6 threshold, and most average variance extracted (AVE) values were above 0.5. For two constructs (job performance and techno-overload), AVE values were slightly below 0.5 but accompanied by high CR (CR = 0.920 and 0.855, respectively), which still indicates acceptable convergent validity, as suggested by Fornell and Larcker^53^ and supported in subsequent partial least square structural equation modeling (PLS-SEM) research emphasizing CR over AVE when factor loadings are acceptable.^54^ Discriminant validity was verified because the square roots of AVEs exceeded the inter-construct correlations, and all correlations were below 0.85. Detailed reliability and validity results are presented in Tables 4 and 5.

**Table 4:** Standardized factor loadings, Cronbach’s alpha (α), composite reliability (CR), and average variance extracted (AVE) (*n* = 400)

Construct	Item	Loadings	α	CR	AVE
Job insecurity (JI)	JI1	0.800	0.845	0.907	0.584
	JI2	0.908	–	–	–
	JI3	0.910	–	–	–
Job satisfaction (JS)	JS1	0.918	0.873	0.922	0.635
	JS2	0.882	–	–	–
	JS3	0.877	–	–	–
Job performance (PR)	PR1	0.828	0.893	0.920	0.484
	PR2	0.815	–	–	–
	PR3	0.800	–	–	–
	PR4	0.878	–	–	–
	PR5	0.848	–	–	–
AI self-esteem threat (SET)	SET1	0.904	0.798	0.882	0.514
	SET2	0.937	–	–	–
	SET3	0.676	–	–	–
AI reliability (RE)	RE1	0.928	0.923	0.950	0.746
	RE2	0.927	–	–	–
	RE3	0.933	–	–	–
AI complexity (TC)	TC1	0.874	0.851	0.910	0.594
	TC2	0.866	–	–	–
	TC3	0.894	–	–	–
AI uncertainty (TU)	TU1	0.772	0.829	0.893	0.543
	TU2	0.914	–	–	–
	TU3	0.884	–	–	–
AI overload (TO)	TO1	0.874	0.744	0.855	0.444
	TO2	0.882	–	–	–
	TO3	0.677	–	–	–

**Table 5:** Correlation results between constructs

	RE	TC	JI	TO	PR	SET	TU	JS
AI reliability (RE)	**0.864**	0.101	0.210	0.026	0.326	0.138	0.478	0.175
AI complexity (TC)	0.101	**0.771**	0.407	0.309	−0.096	0.370	0.239	−0.089
Job insecurity (JI)	0.210	0.407	**0.764**	0.408	0.023	0.607	0.237	−0.162
AI overload (TO)	0.026	0.309	0.408	**0.666**	0.049	0.381	0.186	−0.112
Job performance (PR)	0.326	−0.096	0.023	0.049	**0.696**	−0.004	0.171	0.472
AI self-esteem threat (SET)	0.138	0.370	0.607	0.381	−0.004	**0.717**	0.226	−0.086
AI uncertainty (TU)	0.478	0.239	0.237	0.186	0.171	0.226	**0.737**	0.115
Job satisfaction (JS)	0.175	−0.089	−0.162	−0.112	0.472	−0.086	0.115	**0.797**

The bold numbers on the leading diagonal elements denote the square root of the average variance extracted (AVE).

### Structural model assessment

**Figure 2:**
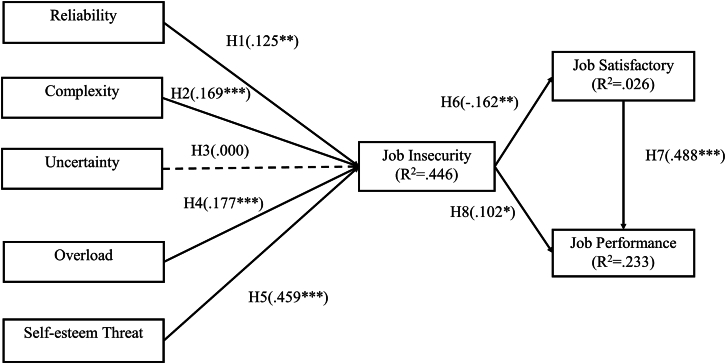
PLS path analysis results Partial least squares structural equation modeling (PLS-SEM) results showing standardized path coefficients (β) and explained variances (R^2^) for the relationships among AI-related technostressors, job insecurity, job satisfaction, and job performance. Significance thresholds, *p* < 0.05 (∗), *p* < 0.01 (∗∗), *p* < 0.001 (∗∗∗).

Figure 2 presents the PLS-SEM results, including standardized path coefficients and explained variances (R^2^) using bootstrapping in SmartPLS. The model accounted for 44.6% of the variance in job insecurity, 2.6% in job satisfaction, and 23.3% in job performance. Among the AI-related stressors, self-esteem threat showed the strongest positive effect on job insecurity (β = 0.459, *p* < 0.001), whereas AI uncertainty was nonsignificant. Path coefficients ranged from 0.125 to 0.459, and all significant relationships were positive. The results of hypothesis testing are summarized in Table 6.

**Table 6:** Hypothesis testing results

Hypothesis	Supported?
H1: AI reliability negatively impacts physicians’ job insecurity.	No (significant but opposite effect)
H2: AI complexity positively impacts physicians’ job insecurity.	Yes
H3: AI uncertainty positively impacts physicians’ job insecurity.	No
H4: AI overload positively impacts physicians’ job insecurity.	Yes
H5: AI self-esteem threat positively impacts physicians’ job insecurity.	Yes
H6: Physicians’ job insecurity negatively impacts job satisfaction.	Yes
H7: Physicians’ job satisfaction positively impacts job performance.	Yes
H8: Physicians’ job insecurity negatively impacts job performance.	No (significant but opposite effect)

Supported at a significance level of 0.05.

## Discussion

### Theoretical contribution

This study extends Califf’s technostress model with perceived AI self-esteem Threat to examine how AI technostress impacts physicians’ job insecurity, satisfaction, and performance. A survey across three Taiwanese hospitals validated the model, revealing unique relationships in medical AI contexts. Integrating self-esteem threat addresses a critical gap, providing new insights into AI-induced stressors’ effects on physicians’ well-being and identity. This is the first empirical study on extended AI-induced technostress and job insecurity from physicians’ perspectives, offering timely contributions to research and practice.

### Technostress and job insecurity: Significant impact with some unexpected findings

This study confirms that technostressors from Califf’s Model—except uncertainty—such as reliability, complexity, and overload, significantly impact physicians’ job insecurity. Adding the AI self-esteem threat construct further revealed its dominant influence.

Notably, PLS path analysis shows that AI reliability positively impacts job insecurity, contrary to the expected direction in Califf’s technostress model, providing partial support for hypothesis H1. Several explanations may account for this finding. Hancock et al.^55^ noted that increased AI reliability can create trust issues in human-AI collaboration, heightening stress and job insecurity. In medical decision-making, physicians may fear that reliable AI systems could diminish their professional value. Wang et al.^56^ and Brougham & Haar^57^ observed that as AI handles complex tasks, employees worry their skills may become obsolete, increasing job insecurity. Role transformations induced by AI reliability further exacerbate these fears.^58^ Additionally, Shoss^59^ highlighted that technological changes closely correlate with job insecurity, as reliable AI systems may intensify concerns about replacement.

We confirmed that AI complexity significantly impacts physicians’ job insecurity, supporting hypothesis H2. High complexity reduces professional confidence and increases insecurity, as physicians struggle to master and trust opaque, unpredictable systems.^60^ Tarafdar et al.^14^ identified technocomplexity as a major source of technostress, where the perceived difficulty in learning and using complex technologies increases stress and workload. Ragu-Nathan et al.^14^ further found that frequent technological changes and uncertainty can reduce users’ confidence and satisfaction, indirectly shaping their job-related attitudes.

PLS path analysis shows that AI uncertainty has a negligible impact on physicians’ job insecurity, rejecting hypothesis H3. This suggests that despite constant advancements in AI technology, physicians do not see these changes as significant threats. They may have adapted to rapid updates or view technological advancements as routine aspects of their work rather than challenges to their professional competencies. We also confirmed that AI overload has a significant positive impact on physicians’ job insecurity, supporting hypothesis H4. Excessive reliance on AI increases workload and stress, heightening concerns about job stability. Tarafdar et al.^37^ noted that unavoidable technological tools in workflows amplify technostress and workload, which in turn contribute to feelings of professional instability and dissatisfaction. Ayyagari et al.^25^ highlighted that excessive information and tool overload make employees feel out of control, which intensifies stress and reduces their sense of autonomy. In the context of AI in healthcare, this overload is particularly relevant as physicians increasingly rely on AI systems for tasks traditionally within their control, which may lead to concerns about reduced decision-making authority and professional independence.^61^ Additionally, managing complex, evolving AI systems can overwhelm physicians, escalating stress and concerns about the impact on their professional roles and future work.^37^

To our best knowledge, this study is among the first to systematically investigate and highlights that AI-induced self-esteem threat is a major source of stress for physicians. The empirical research confirmed its significant positive impact on job insecurity in medical AI contexts (path coefficient 0.459), thus supporting hypothesis H5. This finding aligns with literature emphasizing self-esteem’s critical role in the workplace, particularly amid rapid AI advancements.

Technology self-esteem threat is a form of technostress, particularly in specialized fields like healthcare, where declining self-efficacy impacts job performance and mental health.^62^^,^^63^ In healthcare, AI advancements can undermine physicians’ self-efficacy, intensifying perceived self-esteem threats. Our study confirms this psychological burden, aligning with Tarafdar et al.,^64^ who identified techno-distress as a manifestation of technostress, involving anxiety, burnout, and depression caused by technology overuse. These emotions stem from challenges in adapting to AI technologies, leading to doubts about abilities and diminished self-esteem. This threat arises not only from fears of technological replacement by AI but also from the perception that professional judgment and status are being undermined as AI becomes more integrated into clinical decision-making.

AI tools are increasingly demonstrating capabilities that rival or exceed human practitioners in clinical predictions and diagnoses. This technological shift fundamentally challenges the physician’s professional competence and established medical authority.^3^ As healthcare laws recognize AI’s benefits and potentially loosen restrictions (e.g., doctor-to-patient ratios), the self-esteem threat posed by AI could further heighten physicians’ job insecurity.

### Job insecurity, satisfaction, and performance: Significant impact with some unexpected findings

Path analysis confirmed that job insecurity negatively impacts physicians’ job satisfaction, supporting hypothesis H6. Jiang and Lavaysse^65^ highlighted a significant negative correlation, as job instability lowers satisfaction by affecting professional identity and accomplishment. Additionally, job insecurity reduces trust in the organization, further decreasing satisfaction.^66^ Shoss^59^ noted that insecurity fosters distrust, weakening employees’ connection to their work and organization.

Physicians’ job insecurity with medical AI arises from fears that AI could replace their skills and judgment, threatening their professional status and value. As AI expands in diagnosis and treatment, physicians may feel their expertise is undermined, reducing confidence, motivation, and engagement. This psychological pressure lowers job satisfaction, weakens identification with their career, and disrupts team collaboration and efficiency.

The PLS path analysis confirms that job satisfaction significantly impacts physicians’ job performance, supporting hypothesis H7. Studies consistently show that satisfied employees are more motivated and engaged, enhancing performance, especially in professional roles like physicians.^63^ Job satisfaction also improves mental health and professional engagement, crucial in high-stress fields like healthcare.^67^ Parker et al.^68^ noted that job design influences satisfaction and performance, boosting creativity and problem-solving. Additionally, Bakker et al.^69^ found that in emotionally demanding fields like healthcare, satisfied physicians deliver higher standards of service and patient care.

The PLS path analysis reveals that job insecurity positively impacts physicians’ job performance, contrary to the negative relationship hypothesized in H8 (partial support). This unique finding in medical AI contexts suggests that job insecurity may prompt self-protective behaviors, such as enhanced performance to demonstrate value and reduce instability. Shoss^59^ noted that job insecurity, while often linked to negative outcomes, can sometimes boost motivation, improving performance. Similarly, Probst^70^ emphasized that insecurity can drive employees to increase productivity to secure their roles, particularly in high-pressure environments like healthcare, leading to short-term performance improvements.

Additionally, Lee et al.^66^ found that job insecurity can temporarily boost performance as employees strive to maintain their job status, though it may lead to burnout over time. Similarly, research suggests that job insecurity can act as a motivator, enhancing focus and efficiency during technological changes.^71^^,^^72^ In competitive environments, job insecurity may drive efforts and performance, especially when employees see improved performance as a way to secure their roles.^73^

This finding suggests that job insecurity can enhance physicians’ work motivation, particularly in medical AI contexts, where they may strive to demonstrate their value and irreplaceability. Physicians might focus on technical learning and decision-making to strengthen their expertise in AI-assisted diagnostics, turning emotional pressure into a driver of efficiency and engagement. However, this response is likely short-term, as prolonged stress requires proper management to prevent adverse effects on mental health.

### Research findings and implications

Key findings indicate associations between AI reliability, complexity, overload, and self-esteem threat with job insecurity, with self-esteem threat having the strongest effect (β = 0.459). Uncertainty showed no significant impact. Job insecurity negatively related job satisfaction but unexpectedly linked to higher job performance, as physicians may work harder to demonstrate irreplaceability. However, this short-term performance increase could lead to long-term stress and burnout. These results offer actionable recommendations for governments, healthcare institutions, and researchers to address these challenges effectively.

Governments should develop policies to mitigate AI’s psychological impact on healthcare workers by ensuring transparency and explainability in AI systems to ease concerns about reliability and self-esteem threats. Funding continuous education programs can improve AI literacy, addressing system complexity and reducing technostress. Collaboration between AI developers and healthcare professionals should be encouraged to design tools that enhance, rather than replace, professional expertise.

Healthcare institutions should reduce AI-induced stressors by providing regular training to ease complexity and overload and aligning AI tools with workflows through cross-departmental teams. Recognizing physicians who effectively use AI can address job insecurity and boost engagement.

Physicians should actively engage in AI training to build confidence and adapt their roles to collaborate with AI while maintaining autonomy. Participating in multidisciplinary teams can help shape AI integration to support their expertise.

For researchers, these findings underscore the need to investigate AI’s psychological and organizational impacts on healthcare professionals. Future studies should examine stressors like self-esteem threat and complexity across roles such as nurses and radiologists. Developing validated tools to measure AI-induced technostress and job insecurity will enable more accurate assessments. Longitudinal research on the interplay between technostress, job satisfaction, and performance can provide deeper insights into AI’s long-term effects.

Recent research has shown that physicians increasingly express anxiety about the evolving role of AI in medicine and the potential devaluation of their professional expertise.^21^^,^^58^ Similarly, medical students have also expressed mixed perceptions regarding AI in clinical education and practice. A survey of 325 medical students reported that while 72.3% believed AI could reduce medical errors, 40.3% worried that it might devalue the medical profession.^74^ These findings indicate that AI integration influences not only current practitioners but also future physicians, extending its psychological impact to the formative stages of professional identity during medical training. Collectively, these patterns highlight that adaptation to AI begins well before clinical practice and may shape how future physicians perceive job insecurity and technological change.

Although this study was conducted in Taiwan, the psychological mechanisms identified—such as AI-induced self-esteem threat, job insecurity, and perceived overload—are likely to be relevant to healthcare professionals globally. Similar patterns of technostress and professional identity challenges have been reported in both Western and Asian healthcare systems undergoing rapid digital transformation. However, cross-national differences in medical hierarchy, technological readiness, and cultural attitudes toward automation may moderate these relationships. Future comparative studies are therefore recommended to validate the model in diverse healthcare contexts and to explore how institutional and cultural factors influence AI-related stress and adaptation.

### Future research

Future research should include additional AI-related stressors, such as trust and explainability, to strengthen explanatory power. Expanding the scope to nurses, radiologists, pharmacists, and other professionals can uncover unique challenges they face with AI integration. Longitudinal studies are needed to assess AI’s long-term effects on mental health, career trajectories, and organizational outcomes like burnout and turnover. Broader research across diverse institutions, countries, and healthcare systems will enhance generalizability and help identify cultural and institutional factors shaping AI-related stress and adaptation. Finally, investigating the stress associated with GAI is essential, given its growing influence in healthcare.

### Limitations of the study

Despite its rigor, this study has limitations. The sample, drawn from three Taiwanese hospitals, may limit generalizability to other institutions or regions, as participation covered approximately half of all physicians. Its questionnaire-based, cross-sectional design captures only immediate AI stressor impacts and relies on self-reported data, which may introduce biases like social desirability or recall bias. Future research should adopt longitudinal and multi-national designs to address these gaps. Another limitation is that we did not examine potential moderating effects of sex/gender, and we did not collect data on ancestry, race, or ethnicity. These sociodemographic factors may influence responses to AI-related technostress and should be explored in future research.

## Resource availability

### Lead contact

Further information and requests for data access should be directed to and will be fulfilled by the lead contact, Chung-Feng Liu (chungfengliu@gmail.com).

### Materials availability

This paper did not generate new unique materials.

### Data and code availability

• The anonymized data utilized in this study are not eligible for deposition in a public repository. However, the dataset can be made available upon reasonable request to the lead contact, adhering to the terms of the IRB and licensing agreements.
• No custom code was used in this study. All analyses were conducted using SmartPLS 4.0 and SPSS Statistics (version 26.0), both of which are graphical user interface-based software tools that do not generate shareable code.
• Any additional information required to reanalyze the data reported in this paper is available from the lead contact upon request.

## Acknowledgments

This work was supported by the 10.13039/100020595National Science and Technology Council of Taiwan (grant number NSTC112-2410-H-384-001-MY2).

## Author contributions

C.-F.L.: conceptualization, funding acquisition, writing – review & editing, writing – original draft, validation, resources, methodology, investigation, formal analysis, and data curation. T.-C.L.: writing – original draft, investigation, validation, formal analysis, and data curation. Y.-L.K.: project administration, formal analysis, and writing – review & editing. All authors have meticulously read and unanimously agreed to the published version of the manuscript.

## Declaration of interests

The authors declare no conflict of interest.

## STAR★Methods

### Key resources table

**Table:** 

REAGENT or RESOURCE	SOURCE	IDENTIFIER
**Deposited data**		

Anonymized physician questionnaire data	Chi Mei Medical Center, Chi Mei Hospital Liouying campus, and Chi Mei Hospital Chiali campus	Controlled access-available from the lead contact upon reasonable request; IRB restrictions apply.

**Software and algorithms**		

SmartPLS 4.0	SmartPLS GmbH	https://www.smartpls.com
SPSS Statistics v26.0	IBM	https://www.ibm.com/support/pages/downloading-ibm-spss-statistics-26-end-support-30-sep-2025

### Experimental model and study participant details

This cross-sectional survey was approved by the Chi Mei Medical Center Institutional Review Board (IRB No.11201-003). Eligible participants were practicing physicians (residents and attending physicians) at three participating hospitals. Questionnaires were distributed between 21 February and 3 June 2024. A total of 433 questionnaires were distributed across the three hospitals. After applying predefined data-quality criteria, 33 questionnaires were excluded (e.g., submitted by non-physicians or with missing construct items), yielding 400 valid anonymous questionnaires for analysis (overall response rate 92.4%). The 400 valid responses comprised 293 from the medical center (293/639,45.9% of medical center physicians), 78 from the regional hospital (78/149,52.3%), and 29 from the district hospital (29/59,49.2%).

Sex/gender and other demographic variables. Participant sex/gender distribution is reported in Table 3. We did not perform gender-stratified or gender-interaction analyses in this study. Data on ancestry, race, and ethnicity were not collected and therefore were not analyzed. We acknowledge that the absence of stratified analyses by sex/gender and the lack of ancestry/race/ethnicity data are limitations and recommend that future research investigate potential effect modification by these sociodemographic factors.

### Method details

#### Sampling and distribution

A convenience sampling strategy was applied. Departmental secretaries distributed questionnaires to approximately 50% of physicians within their departments. This sampling ratio was determined by budget considerations and by the sample size requirements suitable for Partial Least Squares Structural Equation Modeling (PLS-SEM) analysis.

#### Consent and incentives

Participants received an information sheet describing the study purpose, confidentiality, and voluntary nature of participation; written signatures were not required. Return of a completed questionnaire was taken as implied consent. A small monetary incentive was provided (∼USD 7) to participants.

#### Research framework and hypotheses

In this study, job performance was defined as the primary outcome, representing the main behavioral manifestation of physicians’ adaptation to AI-related technostress. Job insecurity and job satisfaction were considered secondary outcomes, reflecting intermediate psychological and attitudinal responses to AI-induced stressors. The independent variables included five AI-related technostressors—AI reliability, AI complexity, AI uncertainty, AI overload, and self-esteem threat—derived from the extended Califf’s Technostress Model.

Based on the insights and findings outlined in the literature review and practical observations, the research framework is proposed as depicted in Figure 1.

We summarize the hypotheses derived from the literature review as follows:

H1: AI reliability negatively impacts physicians’ job insecurity.

H2: AI complexity positively impacts physicians’ job insecurity.

H3: AI uncertainty positively impacts physicians’ job insecurity.

H4: AI overload positively impacts physicians’ job insecurity.

H5: AI self-esteem threat positively impacts physicians’ job insecurity.

H6: Physicians’ job insecurity negatively impacts job satisfaction.

H7: Physicians’ job satisfaction positively impacts job performance.

H8: Physicians’ job insecurity negatively impacts job performance.

The conceptual model and full hypothesis development are provided in Methods S1 (Extended Literature Review and Hypothesis Development) in the supplemental information.

#### Definitions of constructs and measurement items

The constructs of this study are based on previously discussed literature. Table 1 outlines the operational definitions of the independent and dependent variables.

Given physicians’ busy schedules, the questionnaire was designed to be concise yet reliable and valid. The items were first derived from a review of relevant literature and adapted to fit the study’s conceptual framework. The draft questionnaire was then reviewed by three experts—an attending physician, an information management professor, and a data scientist—to evaluate content relevance and clarity. The Content Validity Index (CVI) was calculated and reached 1.0, indicating excellent content validity.^75^ After expert validation, three physicians completed a pilot test to assess clarity and wording. Minor linguistic adjustments were made based on their feedback before finalizing the instrument for formal distribution across participating hospitals. The items were designed to measure each construct, whereas the hypotheses (H1–H8) specify the structural relationships among constructs that were tested using PLS-SEM. Table 2 lists the measurement items and their corresponding references. Each item was measured on a 5-point Likert scale, ranging from 1 (Strongly Disagree) to 5 (Strongly Agree).

#### Statistical analysis

All statistical analyses were conducted using IBM SPSS Statistics (version 26.0) and SmartPLS 4.0.^76^

The measurement model was evaluated for content validity, reliability, convergent validity, and discriminant validity. Content validity was verified through expert review by three domain specialists, and the item-level Content Validity Index (CVI) reached 1.0, indicating full agreement on item relevance and clarity.^75^ Reliability was assessed using Cronbach’s alpha (α > 0.7) and composite reliability (CR > 0.7).^54^^,^^77^ Convergent validity was confirmed when factor loadings exceeded 0.6 and the average variance extracted (AVE) values were above 0.5.^53^ Discriminant validity was established when inter-construct correlations were below 0.85 and the square root of each construct’s AVE exceeded its correlations with other constructs.^54^

The structural model was analyzed using Partial Least Squares Structural Equation Modeling (PLS-SEM) implemented in SmartPLS 4.0. This approach was selected because it is suitable for models involving multiple latent constructs, moderate sample sizes, and non-normal data distributions.^78^ Path coefficients and explanatory power (R^2^) were estimated through a bootstrapping procedure with 5,000 resamples, and statistical significance was evaluated at *p* < 0.05, *p* < 0.01, and *p* < 0.001.

### Quantification and statistical analysis

All statistical analyses were performed using SmartPLS 4.0 and IBM SPSS Statistics version 26.0, both graphical user–interface software programs that do not generate shareable code.

#### Measurement model evaluation

Internal consistency reliability was assessed using Cronbach’s alpha and composite reliability (CR); both exceeded the recommended threshold of 0.70 for all constructs. Convergent validity was evaluated using standardized factor loadings and average variance extracted (AVE). All loadings ranged from 0.676 to 0.937, exceeding the 0.60 criterion, and AVE values met or exceeded 0.50 for most constructs. For Job Performance and Techno-overload, AVE values were slightly below 0.50 but accompanied by high CR values (0.920 and 0.855), which is considered acceptable based on Fornell–Larcker guidelines. Discriminant validity was confirmed because all inter-construct correlations were <0.85 and the square roots of AVE exceeded the correlations among constructs.

#### Structural model evaluation

The structural model was analyzed using Partial Least Squares Structural Equation Modeling (PLS-SEM) with a bootstrapping procedure of 5,000 resamples. Standardized path coefficients (β), t-values, and exact two-tailed *p*-values were computed to assess statistical significance. Path coefficients ranged from 0.125 to 0.459. Significance thresholds were set at *p* < 0.05, *p* < 0.01, and *p* < 0.001.

The model explained 44.6% of the variance in job insecurity (R^2^ = 0.446), 2.6% in job satisfaction (R^2^ = 0.026), and 23.3% in job performance (R^2^ = 0.233).

#### Reporting of statistical information

For each hypothesized path, statistical results including standardized coefficient (β), t-value, *p*-value, and explained variance (R^2^) are provided in the main text (results section) and denoted in Figure 2: PLS path analysis results. Because the study is based on PLS-SEM, degrees of freedom (df) are not applicable. No additional data transformations were applied.

#### Sample size and missing data

Of 433 questionnaires distributed across the three hospitals, 400 valid anonymous responses were retained for analysis after excluding 33 questionnaires that failed predefined data-quality checks (e.g., non-physician responses or missing core construct items). Missing data among the retained cases were minimal (overall item nonresponse <5%).
