# Supplementary material for: Exploring the impact of AI technostress on physicians’ job insecurity and performance from an empirical multi-hospital study
Source: iScience. 2025 Dec 9;29(1):114394. doi: 10.1016/j.isci.2025.114394 (PMC12811485; doi:10.1016/j.isci.2025.114394)
Supplement: Document S1. Methods S1 [file mmc1.pdf]

iScience, Volume *29*

Supplemental information

Exploring the impact of AI technostress on physicians' job insecurity and performance from an empirical multi-hospital study

Chung-Feng Liu, Tzu-Chi Lin, and Yen-Ling Ko

Methods S1: Extended Literature Review and Hypothesis Development

The literature review initially focused on the technostress framework, identifying Califf et al.'s model as the theoretical foundation. Guided by this model, additional searches were performed in Scopus, Web of Science, and PubMed to locate studies addressing each relevant dimension. Only representative peer-reviewed English-language studies were included. This review was not conducted as a systematic review but rather as a focused, theory-driven narrative review, aiming to identify key theoretical and empirical works sufficient to support the development of the research framework.

AI Application in Healthcare and Its Psychological Challenge

Public health and epidemiology research often uses prospective designs to compare groups or retrospective analyses of historical records to identify risk factors and outcomes. While valuable for population-level insights, these methods often overlook individual patient variability.

AI is transforming healthcare by revolutionizing disease prediction, diagnostics, and personalized treatment through patient-specific big data—such as age, gender, lab results, and medical history—to tailor strategies. With superior predictive capabilities over traditional methods, AI and machine learning enable personalized approaches [22] and support Shared Decision Making (SDM) [23].

AI/ML technologies in healthcare focus on medical imaging and disease prediction using large-scale records. Examples include predicting ICU ventilator weaning timing [24], detecting pulmonary adenocarcinoma with medical images [25], and identifying negation and speculation in radiology reports [26]. The year 2023 marked the explosive growth of large language models (LLMs) with the launch of OpenAI's ChatGPT, which captured global attention due to its outstanding performance. They have been widely adopted across various industries, including healthcare, where they significantly enhance the efficiency of healthcare professionals and reduce their workload [27].

AI in healthcare has advanced rapidly, often outperforming physicians. For example, AI models for shoulder fractures matched specialists in accuracy [28]. An AI pneumonia prediction system in a Taiwan emergency department reduced sepsis shock in pneumonia patients [11]. These advancements have fueled debates on whether AI might replace healthcare professionals in the future [e.g., 7,29].

Despite the significant potential of AI in healthcare, its impact on healthcare professionals warrants careful consideration, as clinical adoption remains limited. A study on AI for pneumonia prediction found significantly fewer AI-assisted cases compared to non-AI-assisted cases in a large hospital. (n=135 vs. n=966) [11]. Another study at the same hospital reported high willingness among physicians to use AI, with an

average score of 4 out of 5 [12]. This highlights the need to continuously evaluate the factors driving or hindering AI adoption and its impact on job security and performance.

Technostress, driven by AI's rapid evolution and complexity, can overwhelm healthcare professionals, causing cognitive overload and anxiety. For instance, physicians using mobile electronic medical records (MEMRs) reported significant technostress due to system complexity [18]. Additionally, AI systems outperforming humans in diagnosis or treatment may undermine healthcare professionals' confidence, creating resistance as they perceive AI as a threat to their expertise and authority.

These challenges underscore AI's impact on medical staff's well-being, job security, and performance. This study explores AI's advancements and pressures on healthcare professionals, aiming to enhance AI integration into clinical practice. Addressing these barriers can unlock AI's potential while minimizing unintended consequences.

Technostress Research Models

Stress is a psychological response to stressors, and excessive technology use can have adverse effects. Technostress was first described by Brod [13] as a "disease of adaptation" caused by the inability to cope with new technologies. Later, Marcoulides [30] noted that ICTs can induce anxiety, extending the concept into empirical research on technology-induced stress. Arnetz & Wiholm [31] define technostress as a state of arousal in computer-dependent employees, and Weil & Rosen [32] include any negative impact of technology on attitudes or behaviors under technostress.

Tarafdar et al. [14] formalized the construct by identifying five core "technostress creators"—techno-overload, techno-invasion, techno-complexity, techno-insecurity, and techno-uncertainty—and demonstrated their negative effects on productivity. Ragu-Nathan et al. [15] empirically validated these stressors, linking them to decreased job satisfaction and organizational commitment. Tarafdar et al. [33] later refined this framework by introducing technostress inhibitors—organizational mechanisms such as literacy facilitation, technical support provision, and involvement facilitation—which can mitigate the negative effects of technostress on productivity. Wang et al. [34] further noted that the impact of these stressors varies across work environments. Ayyagari et al. [35] introduced the Person-Technology Fit Model [36] to study the relationship between technology and stressors, while a survey found 58.6% of government employees experienced technostress [37].

More recently, Califf et al. [16] advanced this concept by highlighting both its positive and negative psychological effects and their impact on professionals' career attitudes in healthcare IT (see Section 2.2.2 for further discussion). With the growing integration of AI into clinical workflows, technostress has further evolved to include AI-related

stressors such as self-esteem threat, which can influence physicians’ emotional well-being, job satisfaction, and job performance.

Califf et al. [16] highlight technostress as the "dark side" of technology but also acknowledge its positive aspects, forming "bright" and "dark" sides. Their integrated model examines technostress's effects on psychological stress, job satisfaction, and performance, with preliminary evidence in healthcare settings.

The model distinguishes positive "challenge technostressors" like usefulness, support, and involvement facilitation, from negative "hindrance technostressors" like unreliability, complexity, uncertainty, insecurity, and overload. These stressors affect job satisfaction, attrition, and turnover intention through psychological reactions.

In Califf et al.’s technostress model, negative psychological reactions arise from technological changes, leading to stress and anxiety. For physicians using AI, job insecurity captures this response, reflecting fears of AI replacing roles and threatening career stability [38]. De Witte [39] further links job insecurity to anxiety and stress, reinforcing its role as a key negative stressor in technostress models. Similarly, Tuan [40] highlights that technostressors like complexity and overload disrupt workflow and heighten job insecurity by hindering adaptation and increasing perceived risks in fast-changing environments. Scholze & Hecker [41] also found that high-reliability technologies reduce psychological stress, enhance trust, and alleviate insecurity, whereas AI overload increases information processing burdens, harms mental health, and amplifies insecurity. In medical settings, perceived AI complexity can increase cognitive demands, while overload from excessive systems amplifies stress. These findings underscore how technological uncertainty and demands contribute to heightened job insecurity.

“Technology insecurity” in Califf et al.’s model refers to technology-induced insecurity, closely resembling "job insecurity," causing conceptual overlap. As a result in this study, job insecurity is treated as the dependent variable, excluding "Technology insecurity" as a technostressor. In healthcare, AI "Reliability" is more nuanced than "Unreliability," as high reliability can also induce stress. Thus, "Reliability" is used as the stressor in the model. Four hypotheses are proposed:

H1: AI reliability negatively impacts physicians' job insecurity.

H2: AI complexity positively impacts physicians' job insecurity.

H3: AI uncertainty positively impacts physicians' job insecurity.

H4: AI overload positively impacts physicians' job insecurity.

Self-esteem reflects an individual's evaluation of their worth, encompassing abilities, achievements, and social image [42]. External challenges can threaten self-worth, causing anxiety and insecurity [43]. Self-esteem threats also exist in the use of technology. The superiority of technology can undermine users' self-esteem, thereby intensifying feelings of insecurity [41]. Smart technologies may enhance performance (bright side) but diminish individual distinctions, threatening self-esteem and job performance [44]. In healthcare, AI's superior performance often challenges physicians' judgments, heightening self-esteem threats among professionals [3].

Dweck & Leggett [45] proposed the social-cognitive theory, suggesting individuals adopt defensive behaviors to protect self-esteem in threatening situations. As AI advances, the workforce faces challenges requiring new skills like technology use and data analysis [46,47]. Physicians' acceptance of clinical IT is often hindered by perceived threats to professional autonomy [48]. AI's rapid progress, potentially surpassing their expertise, triggers defensive responses and job insecurity, increasing resistance to adopting these technologies in clinical settings. Therefore, we hypothesize:

H5. AI self-esteem threat positively impacts physicians' job insecurity.

Califf et al.’s Technostress Model highlights psychological responses to technology but overlooks self-esteem threats, a crucial factor in high-stakes fields like healthcare. This study incorporates self-esteem threat into the model to better understand the psychological impacts of AI adoption in medicine.

Job Insecurity, Job Satisfaction, and Job Performance

Califf’s technostress model suggests negative psychological responses can impact job satisfaction and turnover intentions. Studies show job insecurity reduces satisfaction and harms performance [49,50,40,41]. Additionally, an increase in job satisfaction is generally believed to enhance work engagement, which in turn improves employee performance [40]. Facing AI, physicians may fear reduced professional value and role replacement, lowering satisfaction and engagement [39]. However, for physicians, job insecurity from AI is less likely to cause quitting but more likely to affect satisfaction and performance. Thus, we suppose that job insecurity influences physician satisfaction and performance rather than directly causing turnover, leading to the following hypotheses:

H6: Physicians’ job insecurity negatively impacts job satisfaction.

H7: Physicians’ job satisfaction positively impacts job performance.

H8: Physicians’ job insecurity negatively impacts job performance.
